# “Just pee in the diaper” - a constructivist grounded theory study of moral distress enabling neglect in nursing homes

**DOI:** 10.1186/s12877-024-04920-7

**Published:** 2024-04-24

**Authors:** Stine Borgen Lund, Wenche K. Malmedal, Laura Mosqueda, John-Arne Skolbekken

**Affiliations:** 1 Department of Public Health and Nursing, Norwegian University of Science and Technology (NTNU), 7491 Trondheim, PO Box 8905, Norway; 2 Department of Family Medicine, Keck School of Medicine, University of Southern California, Los Angeles, USA

**Keywords:** Moral distress, Neglect, Missed care, Coping, Nursing home, Long-term care, Constructivist grounded theory

## Abstract

**Background:**

A growing body of evidence shows that many nursing home residents’ basic care needs are neglected, and residents do not receive qualitatively good care. This neglect challenges nursing staff´s professional and personal ideals and standards for care and may contribute to moral distress. The aim of this study was to investigate how nursing staff manage being a part of a neglectful work culture, based on the research question: “How do nursing home staff manage their moral distress related to neglectful care practices?”

**Methods:**

A qualitative design was chosen, guided by Charmaz´s constructivist grounded theory. The study was based on 10 individual interviews and five focus group discussions (30 participants in total) with nursing home staff working in 17 different nursing homes in Norway.

**Results:**

Nursing staff strive to manage their moral distress related to neglectful care practices in different ways: by favouring efficiency and tolerating neglect they adapt to and accept these care practices. By disengaging emotionally and retreating physically from care they avoid confronting morally distressing situations. These approaches may temporarily mitigate the moral distress of nursing staff, whilst also creating a staff-centred and self-protecting work culture enabling neglect in nursing homes.

**Conclusions:**

Our findings represent a shift from a resident-centred to a staff-centred work culture, whereby the nursing staff use self-protecting strategies to make their workday manageable and liveable. This strongly indicates a compromise in the quality of care that enables the continuation of neglectful care practices in Norwegian nursing homes. Finding ways of breaking a downward spiralling quality of care are thus a major concern following our findings.

**Supplementary Information:**

The online version contains supplementary material available at 10.1186/s12877-024-04920-7.

## Background

A growing body of evidence shows that basic care needs of nursing home residents are regularly neglected, and residents do not always receive qualitatively good care of basic human needs. Hence, these neglectful practices may include not providing sufficient basic care or ignoring residents’ needs related to nursing home residents’ physical, psychological, emotional, and social needs. Examples of this include omitting mouthcare on a regular basis, ignoring residents with challenging or aggressive behaviour, and lack of attention to a residents’ need for social stimuli. The literature presents different perspectives of what constitutes neglect of nursing home residents’ basic needs. In this paper we use neglective care practices given examples of above to address these practices regardless of the perspective taken [1–7].

Not being able to provide sufficient care or observing colleagues providing compromised quality of care is found to be a major stressor for nursing staff [8–12]. This may lead to physiological and emotional stress [10], compassion fatigue [13], troubled conscience [14] and stress of conscience [8], among other forms of pressure, all of which may potentially result in moral distress [9, 11, 15–17]. Moral distress has been recognised as a major problem for health care staff in all care systems for over four decades [18, 19]. The concept of moral distress was introduced by the philosopher Andrew Jameton in 1984 and has been further developed and enhanced by him and other scholars in recent decades [18]. We lean toward Nathaniel’s definition of moral distress based on a synthesis of previous definitions by Jameton (1984), Wilkinson (1987-88) and Nathaniel (2004):

“Moral distress is pain affecting the mind, the body, or relationships that results from a patient care situation in which the nurse is aware of a moral problem, acknowledges moral responsibility, and makes a moral judgment about the correct action, yet, as a result of real or perceived constraints, participates, either by act or omission, in a manner he or she perceives to be morally wrong” (p. 421) [20].

Moral distress occurs when nurses or other health care staff are unable to act in accordance with their personal values or/and professional judgement when it comes to external constraints, such as lack of resources, or internal characteristics related to moral judgement [21].There is a high prevalence of moral distress in caring for people with dementia [16, 22]. However, knowledge about moral distress in nursing homes in general is limited, and few studies relate this to compromised quality of care [9, 11, 16, 17, 22]. Organisational, institutional or structural constraints, such as a lack of resources, which may contribute to compromised quality of care and suffering residents, are among the main reasons for moral distress among nursing home staff [9, 11, 16]. In addition, individual and cultural obstacles like having to act in contradiction to personal knowledge, beliefs or values is a major stressor [9]. Nursing staff may not only be troubled by what they have done, but also by what they have not done or should have done [15].

Moral distress affect nursing staff negatively both psychologically and physically [23]. Not being able to provide care, or providing compromised quality to the elderly contributes to staff reports of feeling emotionally drained or physically exhausted [16]. This may lead to feelings of inadequacy, frustration, anger, powerlessness, helpless, heavy or troubled conscience, sadness, guilt and shame [9, 11, 14, 22, 24], which over time can increase the risk of a person becoming cynical, bitter, callous and resigned [25]. Physical symptoms of moral distress include fatigue, exhaustion, headaches, stomach pain, and sleeplessness [15]. Furthermore, moral distress in nursing homes is associated with illness, decreased job-satisfaction, risk of burn-out, absence from work and increased intention to leave– all with the potentially negative impact on the quality of care [11, 25–27].

Different ways of handling moral distress are presented in the literature, describing possible responses by caregivers to avoid or combat their moral distress: to acquiesce, maintaining a lack of awareness, to withdraw from distressing situations, to fight, or to reach a satisfactory resolution [23, 28]. Cognitive dissonance reduction strategies are other ways caregivers handle moral distress. This can mitigate against distress through three different approaches: changing one’s appraisal, minimising the importance of dissonant thoughts, or creating new congruent ones [10]. A theory of conformity has also been developed, whereby beliefs, attitudes and behaviours corresponding to group norms [29] are nurtured as a way to manage moral distress related to providing substandard care [30].

To our knowledge, no studies have explored the consequences of being a part of a neglectful work culture on nursing home staff, and how individuals manage this. In accordance with the constructivist grounded theory (CGT) approach guiding our work [31], we have sought to understand the processes influencing a neglectful work culture in nursing homes. We wanted to investigate the social influences that shape staff members’ experiences of neglectful care practices and responses to them, focusing on the relation to moral distress. In this study we identify processes that shape staff members’ perceptions, experiences, and responses regarding neglectful care practices, and their relation to moral distress. The aim of this study was to investigate how nursing staff manage being a part of a neglectful work culture, based on the research question: “How do nursing home staff manage their moral distress related to neglectful care practices?”

### Structure and organisation of Norwegian nursing homes

Norwegian nursing homes are 24- hours skilled nursing facilities providing a level of care between specialized care sector, such as hospital and home-based care. The average size of nursing homes in Norway is over 50 beds, but this varies considerably [32]. The mean age for residents in Norwegian nursing homes is 85 years, and severe and complex comorbidities are highly prevalent. Consequently, polypharmacy is also common, requiring close follow-up, supervision, and support in activities of daily living. Almost 8 to 10% have dementia with accompanying neuropsychiatric symptoms such as agitation, aggression, anxiety, and depression [33].

Norwegian nursing home care is delivered under the National Regulation of Quality of Care to ensure that residents’ basic needs including physical, psychological, and social needs are met, in addition to respect, security and independence [32, 34]. The Ministry of Health and Care Services launched the Dignity Guarantee for elder persons in 2010, where healthcare services should work towards a “dignified, safe and meaningful life” for older persons [35]. Norwegian nursing homes strive to promote resident-centred care (RCC) to meet the existing quality standards for care [36]. RCC is influenced by a person-centred care (PCC) first introduced by Kitwood in 1997, and decades of practice and research confirms that person-centred care has become the gold standard to strive for in long-term care and dementia care. RCC facilitates a holistic view of the resident, recognising residents’ preferences and values, promotes autonomy, and right to self-determination. RCC emphasises partnerships between the health- carer and resident, in addition to care flexibility in attempt to contribute to meaningful lives and promote well-being for the residents [37–39].

There is no mandatary staff-to resident ratio, standards for nursing staff´s qualifications or requirements regarding skill-mix [40]. A high number of unskilled personnel are hired due to a shortage of registered nurses (RN), recruitment problems, and challenges of keeping nurses (RN) in nursing homes. Norwegian nursing homes are characterised by high physical and psychological workload and time pressure, high turnover among RN´s and licenced practical nurses (LPN´s), lack of competent personnel, high absence from work and intention to leave, all of which have a negative effect on quality of care [26, 41].

## Method

Based on the aim and research question, qualitative method and research design was found appropriate. We chose to use constructivist grounded theory (CGT) approach and interviewed nursing homes staff with experience in direct resident care in nursing homes.

### Constructivist grounded theory

CGT is a contemporary version of Grounded theory emanating from the idea that interactions between people create new insights and knowledge, acknowledges multiple realities, and underpins how participants construct meaning in the relation to the area of inquiry. CGT locates the research process and product in historical, situational, and social conditions. It is both flexible and structured, uses constant comparisons and provide tools for constructing theory. CGT requires simultaneous data-collection and analysis, performed in an iterative process. Rather than discovering theory the researcher is constructing theory [31]. Our research team consists of researchers from different professions and disciplines; the first author SBL is a Critical care nurse with decades of clinical and research experience in neurocritical care, with patients having severe cognitive deficits and challenges. The co-authors are all professors; JAS is a licenced psychologist with decades of experience in qualitative research. WKM is a registered nurse and LM is a Doctor of Medicine, both have long experience in the research field of elder abuse and neglect. Our diverse research backgrounds and experiences provide a broader perspective on the theme and possibilities for richer interpretations of our results.

### Research design

For this study, data were gathered through a combination of focus-group (FG) discussions and individual interviews. We initially chose focus groups for their potential to provide insights into specific themes as well as to produce rich data, and of logistic reasons making the data collection doable. In addition, group processes can produce a synergistic effect, potentially creating new knowledge and perspectives [42]. Focus groups are used to find a range of reflections of people across several groups. It also is suitable in studies wanting to explore experiences, attitudes, and how knowledge is produces and used in a particular cultural context- such as working in a neglectful work culture. As insufficiencies in care provision can be a sensitive topic to discuss openly in group interactions, we supplemented the focus groups with individual interviews.

### Sampling

We started initial sampling by strategically selecting nursing homes (long-term care facilities) in an urban city in mid-Norway. Nursing care staff was recruited through an information letter about the study distributed via the nursing home management. In addition, other participants were reached via the online information-channel for nursing students, and a practice seminar where the first author was lecturing about elder abuse and neglect in nursing homes. After initial sampling and data analysis of FG discussions, we purposely conducted individual interviews to see if this enrichened and deepened the emergent sub-categories [43, 44]. As the study evolved and the categories becomes more conceptual, we continued with theoretical sampling which was more focused and directed to specific participants. Thus, we wanted to include participants which might voice other perspectives in attempt to provide more variation in the sample. Hence, three participants that had left nursing home practice for conscientious reasons were reached through colleagues asking them to participate.

### Participants

Participants were recruited over a 19-month period from April 2019 to November 2020. From March 2020, the Covid-19 pandemic interfered with our recruitment, and nursing home staff were not easily available in this period. Five FG discussions (with respectively 3, 4, 4, 5, 4 participants) were held and 10 individual interviews were conducted. A total of 30 nursing home staff (27 females, three males; ages: 22–62 years; work experience in nursing homes: 1–28 years) from 17 different nursing homes (four rural, 13 urban) from municipalities in central Norway participated. Only nursing staff with experience in providing direct care to residents in long-term nursing homes were included. Four of the participants were invited for member- checking, and two accepted to be contacted via telephone. The sample included 13 registered nurses (RN), 12 licensed practical nurses (LNP), one social worker (SW), one social educator (SE) and three nurse assistants (A). A more detailed overview of participants has been given elsewhere [45].

### Data collection

We used a semi-structured interview guide, which was developed and adjusted in accordance with our analyses (Additional file 1). After initial analyses, we supplemented FG discussions with individual interviews to provide further insight into this possibly sensitive theme. After the participants’ spontaneous responses had been explored, we introduced case descriptions and examples of neglect from a survey instrument on elderly abuse [2], which led to the development of additional categories. The discussions/interviews lasted 60–90 min and were digitally recorded and transcribed verbatim by a skilled transcriber (HF). This study was conducted according to the guidelines of the Declaration of Helsinki and approved by The Norwegian Centre for Research Data (NSD) (protocol code 221,320, approved 26.02.2019). We used the COREQ checklist to ensure methodological quality (Additional file 2) [46].

### Data analysis

The CGT framework guided this process, involving initial, focused and theoretical coding [31]. The first author performed the initial analyses using line-by-line coding with pen and paper. In addition, the first four FG discussions were coded by the second and last author to ensure credibility. The most frequent initial codes were tested amongst large segments of data, and those codes showing most analytical strength were raised to tentative categories. Focused coding enabled sorting, synthesizing and conceptualizing data, and transformed the fractured data from the initial coding process back to more abstract concepts, thus were beneficial in raising the analytical level. To identify consistencies and differences in the data, we used constant comparisons, continually refining concepts and relevant theoretical categories. This process enabled identification of sub-categories and core categories. Theoretical coding was then used to theorize the data and focused codes, and the codes selected in focused coding were enhanced to more abstraction and formation of a core category. The analysis was done iteratively, moving back and forth between coding the data-material and reading relevant literature and theory in this stage and was continuously redefining tentative categories.

After the five FG discussions and 10 individual interviews, further data collection from participants did not create new properties or provide further insight into our categories. We then carried out member-checking by telephone, which involved taking back our tentative ideas and categories for confirmation, to check and refine our categories. These calls confirmed our tentative sub-categories and categories and supported our core category, and we concluded that sufficient saturation was reached [31, 47].

Field reports and memos were used to provide an audit trail during the data-collection and analysis phases. Field reports were written immediately after each interview containing an overview of the context, participants, and major themes. Analytical memos were written from the early phases of the data-collection and analysis; when reading through the transcripts for the first time and during initial coding and was helpful in questioning and exploring tacit and more explicit treads in the data. Memo-writing was an effective way to conceptualize early data to codes and raise initial codes to more abstract and focused codes. The growing memo-library provided a detailed record of thoughts, ideas, reflections and interpretations during the analytic process. NVivo software version 20 was used to assist the data organisation and coding process.

## Results

**Fig. 1:**
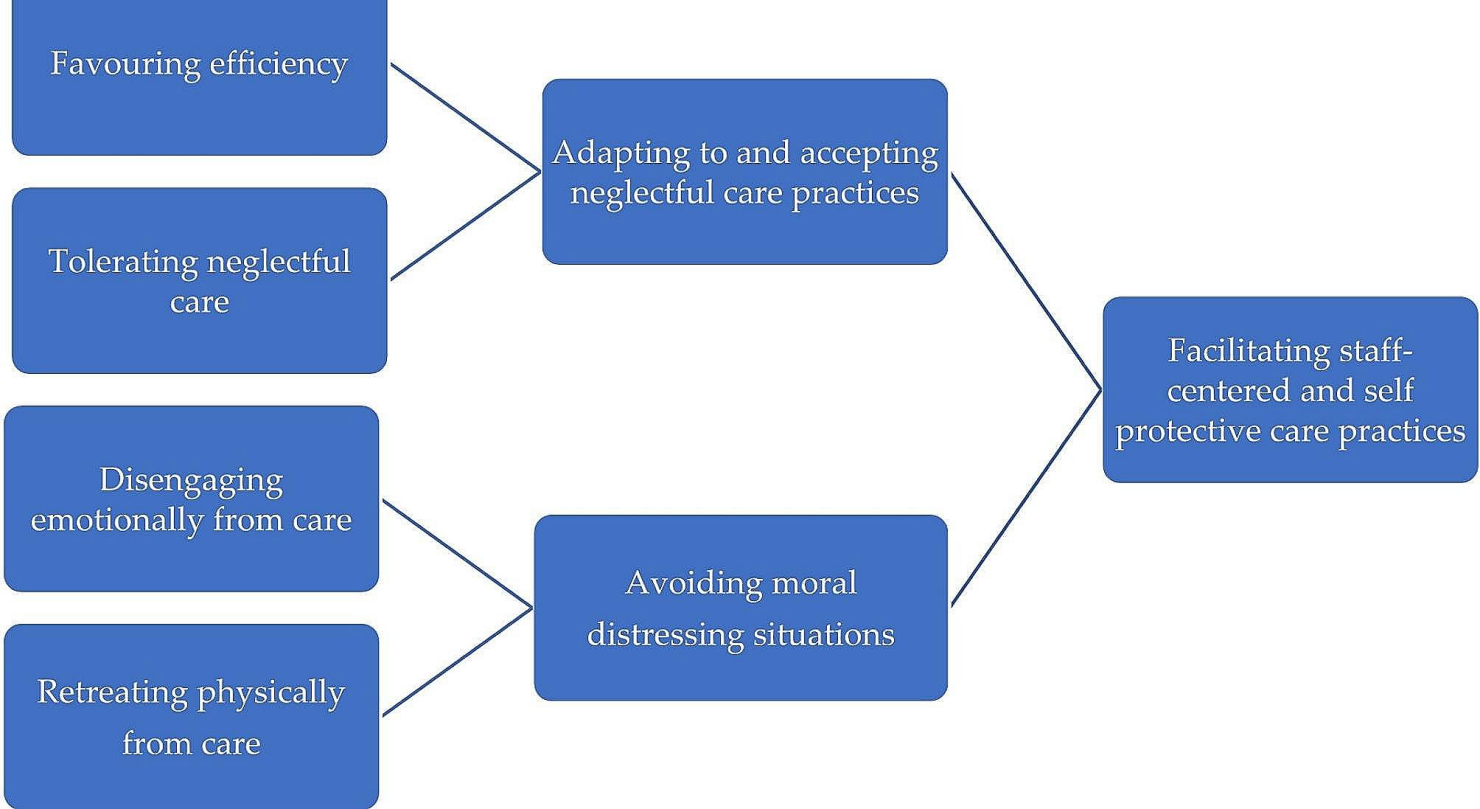
Illustration of the relationships between sub-categories, categories and core category

Our core category is that nursing home staff facilitate staff-centred and self-protective care practices to mitigate their moral distress related to neglectful care practices, as illustrated in Fig. 1.

### Adapting to and accepting neglectful care practices

When faced with moral distress related to neglect, nursing staff responded by adapting to and accepting neglectful care practices through favouring efficiency and tolerating neglectful care.

#### Favouring efficiency

Staff wanted to provide care in accordance with professional and personal nursing ideals that promote residents´ integrity, dignity, and autonomy, as well as spending time to build trusting relations and provide resident-centred care. However, the existing work culture in many nursing homes, and a discrepancy between resources and demands, created a conflict between the ideals and the reality of care provision. This induced staff to compromise on their personal standards of care provision, creating feelings of insufficiency, frustration and despair and leading to increased moral distress.

*And– how easy it is to– if it has been very busy times– to put on a slightly bigger diaper because we may not have time go to the bathroom. It’s terrible, but it happens. Unfortunately. (FI, RN 1)*

One way to manage their moral distress related to neglectful care practices or not being able to meet residents basic care needs was to increase the efficiency of care. By rigidly following routines the nursing staff were able to do work more effectively and faster, which enabled them to cover more of the residents’ basic care needs. Institutional routines were well established among both staff and the residents, and followed without hesitation and staff did not reflect much on this practice. Putting residents to bed early in the afternoon before the night shift due to limited availability of staff at night or waking them up to provide morning care before a hectic day shift, enabled provision of basic physical care. Despite this, few questions were raised about institutional routines. Some respondents reflected that such routines were mainly for the staff’s convenience, whilst also being contradictory to the resident’s wishes.

Another way of meeting excessive care demand was through being more task-oriented and providing standardised care at a high tempo. However, this frequently led to omission or neglect of more time-consuming activities, as well as less visible duties, such as putting lotion on fragile skin or mouth-care. There were examples of staff becoming emotionally “blunted” when adapting to time limitations and cutting corners on a regular basis to maintain efficiency. Providing basic care then became just another task to tick off the list, and to some the nursing home appeared more like a “care factory” where residents became just another task to be performed as quickly as possible, resulting in their objectification and depersonalisation.

*I accept that things go faster, if you see what I mean? At first, I thought it was strange to observe because I felt “damn, what outlook on humanity do you have?” You work with someone who just rushes off. Care activities [e.g. morning care] should be something good and nice, but it goes by so fast that it can look like it’s just a matter of finishing a package… (II, LPN 11)*.

Although working efficiently and constantly, staff still struggled to meet the never-ending care demands. 

This resulted in need of prioritised care, leaving little time for chatting or meeting residents´ broader psychosocial needs. Basic physical care was also regularly missed or delayed, especially in the evening and weekends when fewer carers were present. This resulted in neglectful care practices which created feelings of guilt and shame in the nursing staff, as staff had to face the realities.

*It was a Saturday morning; I was ashamed because she [an older resident] had… a daughter [who] came to visit at 12 o’clock and she still hadn’t had her morning routine, and it wasn’t because we had taken a coffee break. And then I felt ashamed, that I simply hadn’t had time to do it, but that’s how it was. (II, SW 1)*

There were examples of staff wanting to provide resident-centred care, who were not willing to let the residents pay the consequences for the lack of resources. However, taking the necessary time to perform personal care, without skipping or rushing a task, resulted in less efficiency, and having to leave unfinished care to the next shift. This was not always endorsed, and the staff had to endure negative feedback and even scolding from colleagues.

This routine- and task-oriented culture favoured staff´s needs to accomplish their never-ending care duties. When adapting to a work culture favouring efficiency, they tended to create a staff-centred work culture at the expense of resident-centred care provision. However, the opposite was experienced in some rare occasions when members of staff challenged these norms, disputing a work culture that puts carers’ needs before those of residents.

#### Tolerating neglectful care

Nursing staff described a workday with never-ending duties constantly stretching their limits. It was difficult, and for some nearly impossible, to perform in a satisfactory way under the existing work conditions. Staff felt they had to be superhuman in order to cover residents’ care needs, something they clearly are not– leading them to face daily neglect of residents and their needs. This experience of being on a “mission impossible” contributed to a pragmatic attitude, whereby staff tolerated and accepted that care was neglected on a regular basis.

*Then you realise that if you’re going to be able to complete it, [the work] then you don’t get time to do what you should have done, and you change [attitudes] whether you want it or not– the view of what you’re doing changes, the boundaries are shifting. (II, LPN 11)*

This sense of resignation was described as a survival mechanism by participants who were pushed beyond the limits of what they were able to perform. Having to leave residents waiting for help to go to the bathroom was normal due to limited time or available staff. Practices such as putting a diaper on a continent resident in case they were unable to follow the resident to the bathroom in time, thus became normalised. The following statement indicating a tolerance for a neglectful care:

*There are some of those who use a diaper that still can tell when they have to go to the toilet to pee, but I have experienced that [they are told] “but you can just pee in the diaper”. (II, A3)*

Increased tolerance for neglect among colleagues was also observed in situations where supporting resident´s autonomy and letting them refuse care were frequently used as plausible reasons for omitting tasks. Cognitive impairment and failing memory became acceptable excuses for omitting basic physical care, or not meeting psychosocial needs. Ageist attitudes among colleagues, such as “they are old and it goes only downhill from here”, also served as an excuse for neglecting care.

*Yes, I think maybe this change starts when you stop trying– that it will be like you convince yourself that this person doesn’t like to shower anyway so she doesn’t even have to be offered it. There’s no point in asking, just leave it. (II, RN 9)*

Increased tolerance of omissions and neglect of care duties was also explained because of shortcomings in the caregivers, relating to lack of education and competence, or staff not being sufficiently trained to recognise and reflect on ethical and moral dilemmas in dementia care. In addition, exhaustion from overwhelming demands, and near burn-out were presented as plausible reasons for the tolerance of neglect that was observed and experienced.

*But I do notice there is a huge difference among people in what they think is important, and how much responsibility they take. I also think that a lot of this is unconscious, or that you don’t see it. Or that you do see it– I’ll do that later– then it will be forgotten. (FG, SE 1)*

There were examples of concerns raised about the effects of the prevailing work culture on the quality of care. Staff struggled when observing colleagues neglecting residents. Some felt they had been forced to lower their own standards of care and developed a bad conscience as a result. Others challenged a work culture tolerating neglect by setting clear standards for the care they expected, thus making themselves and colleagues responsible and accountable for their care provision. This enabled labelling the omission of care as neglectful, which was not always popular among colleagues.

*But I– we have to talk about this too, you know, that I am aware that if we don’t turn that person now– and a pressure ulcer occurs, it is our fault, I usually say (FG, RN 11).*

An increased tolerance for neglectful care practices was partly a response to challenging work-conditions, which were met with pragmatism and other techniques for handling the huge workload, thus promoting staff-centred care practices. Not all staff members blamed themselves for this neglectful care. Instead, they blamed a system that did not allocate sufficient resources. They argued that existing working conditions put staff in a position that made it nearly impossible to provide good care, forcing them to accept neglectful practices.

*I’ve been in quite a few situations like this myself where you see that things don’t work out in practice, you can’t run fast enough, you don’t have enough arms to do things, and then you don’t feel like it’s your fault. (II, RN 9)*

A work culture tolerating neglect made work more bearable for nursing staff under demanding circumstances. By contrast, managing moral distress related to neglectful care practices in constructive ways typically only took place when staff recognized it and refused to be part of a neglectful work culture. This occurred when staff acknowledged themselves and colleagues as responsible and accountable for their lack of sufficient care.

### Avoiding morally distressing situations

Nursing staff applied two different approaches to distance and protect themselves from the moral distress related to neglectful care: disengaging emotionally from care and retreating physically from care.

#### Disengaging emotionally from care

Participants reported once being eager to care for the residents, but this changed and instead they began to dread going to work. Physical and emotional exhaustion was common after many years of working in nursing homes. Despite taking professional pride in their work, and being satisfied with caring for the elderly, they frequently questioned their ability and motivation for continuing working until retirement under the existing conditions.

*No, the main thing for me is that I feel very constrained as a nurse, I do take pride in performing a qualitatively good job. It makes me despair, and I’m really looking forward to retirement, because I think the squeeze is only getting worse. (II, RN 13)*

Realising that they work with carers who do not want to provide good quality care, or cannot be bothered to, was an important source of moral distress. This was exemplified by situations where colleagues had been found asleep in resident rooms or pretended to have provided care when they obviously had not. When trying to raise their concerns regarding neglectful care practices or inviting colleagues to reflect on their duty to provide care, this was not always endorsed or welcomed by colleagues. Limited time was often given as an excuse by colleagues for not engaging in these staff conversations, but lack of interest and willingness to engage in reflective conversations were also observed.

*But, in a way you don’t have the will to want to do a good job, it may be why… We are different, some want to do a good job, some want to do a very good job, some want to do a bad job, and some want to do a medium good job. All those shadings exist. (FI 5, RN 11)*

Participants further presented disengaging from resident care as a way of protecting themselves from the experience of not being able to meet the care demands. Turning to more pragmatic attitudes to resident-centred care, and lowering standards of care were ways to deal with the resulting moral distress.

*You can change over time and get such a view [reductionist view of people] gradually if you feel that it’s what is needed to get the job done in the hours you are present. (II, LPN 11)*

They struggled with moral distress when observing colleagues or noticing that they themselves disengaged from care, frequently acknowledged this to protect themselves from the strain of being a part of a neglectful work culture. Nevertheless, they also questioned the negative effect this disengagement may have on residents, as well as on their own professional ideals, integrity, and self-esteem. Some longed for a work culture where reflections and discussions about insufficient care practices were welcomed.

#### Retreating physically from care

Staff referred to episodes where they or their colleagues avoided and retreated from care provision, implicitly or explicitly. Explicit examples included colleagues avoiding or ignoring difficult residents: for instance, not answering the bell when a person calls for assistance, instead letting someone else respond. Spending time on private mobile phones or drinking coffee with colleagues instead of engaging with residents were also cited by respondents as ways of distancing themselves from care.

…*those who had the least stressful time at work was those who just didn’t care, because they persevere then. They endure year after year after year. It is those who are constantly trying to reach the goals all the time who quit. (II, LPN 10)*

When staff tried to express the stresses and burden that poor resourcing has placed on care provision, managers typically offered courses in stress-management, or encouraged staff to step down to part-time work. These offers changed the focus from a problematic system to the individual carer, increasing feelings of guilt and self-blame for not managing their present work conditions.

*So, I feel I’m getting sick because it’s about the system, it’s about how we’re treated, but it’s the individuals who will be taken. “You can’t do it [work full-time], what’s wrong with you? What’s wrong with you since you can’t do it?” (II, RN 13)*.

Not all nursing staff members accepted existing conditions but chose to leave the nursing home and even the profession. This sometimes originated from a wish to protect themselves and their own physical and mental health. By leaving the nursing home they also protected the resident from their own neglectful care provision. They were thus neither willing to be part of a work culture that undermines their professional and personal values, nor be faced with a troubled conscience and moral distress on a regular basis.

*I still did a good job. But I gradually started to stop caring. I can’t do that. It’s people I am dealing with. (II, LPN 10)*

Another reason for leaving or considering leaving their job was a feeling of losing hope for a better future. Participants felt they were experiencing a downward spiral with dwindling available resources failing to meet increasing demands; they became deeply concerned about the effect this may have on care provision. Despite these issues, staff still found providing care to residents meaningful and fulfilling. They still questioned how long it will be before neglectful care practices results in serious resident harm or death– and concluded that they were not willing to be a part of these scenarios. By retreating from care, they were protecting residents and themselves from future unavoidable harm.

## Discussion

Nursing home staff find their work very meaningful and as having high standards for care provision and wanting to provide resident-centred care. However, existing work conditions and a neglectful work culture create a conflict between their ideals and the reality of care provision. Consequently, nursing staff find themselves becoming a part of a work culture challenging their professional and personal standards, and contributing to moral distress.

Our main findings are that participants acknowledge facilitating staff-centred and self-protecting care strategies to alleviate moral distress related to being a part of a neglectful work culture. These responses compromise the quality of care and enable the continuation of neglect in nursing homes.

### Facilitating staff-centred care by adapting to and accepting neglect

To alleviate their moral distress, nursing home staff justify their practices by favouring efficiency to complete their care duties in sufficient time. This is a familiar approach, as the work culture in nursing homes traditionally promotes a strong focus on delivering routine physical care and completing task-based work efficiently and quickly [48, 49]. This approach may resolve feelings of moral distress by achieving what appears to be a satisfactory resolution as basic (physical) care is provided [23, 28]. Hence, the nursing staff can achieve a (temporarily) mitigation of their feelings of guilt, shame and frustration when resources and demands mismatches. However, while in the past Norwegian nursing home residents were typically frail and mostly bed-dependent, they are presently recognised as having complex medical conditions, cognitive deficits, and/or psychiatric illness, and challenging behaviours such as agitation and aggression [33]. For this patient population, the availability of skilled staff with sufficient time for holistic care provision is crucial for sufficient quality of care.

Our study participants experienced meeting residents’ complex care needs when constantly pulled between “task and time” as challenging, which is confirmed in prior research [49]. Favouring efficiency to get the job done makes the nursing staff´s workday liveable. This is in accordance with research demonstrating that nursing staff tend to reconcile their expectations for care as a way of adapting to the work culture, minimising their exposure to personal harm [50]. Despite this emphasis on efficient, routine- and task-oriented provision of care, although intended to counter neglect, it nevertheless serves to promote neglectful care practices.

When staff adapt to the mismatch between resources and demands by working faster and in a more standardised way, care provision becomes quick and efficient, but also uncaring and dehumanizing [50, 51]. This leaves little room for individualised and resident-centred care which is the gold standard for high quality of care for nursing home residents [9, 22, 37, 38, 52, 53]. There may be limited opportunities for supporting and stimulating residents´ self-caring abilities, which further exacerbates functional and cognitive decline [49, 54, 55]. Favouring efficiency is a problem-focused coping strategy aimed at solving neglectful care practices in nursing homes by regularising and normalising them. When staff are compromising nursing values and lower care standards to maintain efficiency, it further aggravates the carers’ moral distress, and a vicious cycle of neglect is established.

Our participants tolerated neglectful care to manage challenging working-conditions, including work overload and limited time for care. Acceptance of a difficult situation that is hard to change, and adapting by changing one’s expectations and behaviours, are well-established coping strategies [56], like when trivializing morally challenging situations to mitigate moral distress [57]. This finding can also be in line with the theory of conformity; tolerance of neglect may be explained by a tendency to conform to existing cultural norms, to minimise cognitive dissonance [30]. Accordingly, simple acquiescence has been demonstrated as a response to moral distress. Nursing staff may be aware of the moral situation creating distress but accept the outcome without objecting [28]. This acceptance may lead to staff becoming resigned, cold or blasé, eventually resulting in compromised quality of care [25, 57, 58]. This is confirmed by our participants, who describe a reductionist care culture illustrated by terms such as *“care-factory”* or *“finishing a packet”* about morning care provision. In addition, the admonition *“just pee in the diaper”* is illustrative of a cultural shift from resident-centred care to care provided at the convenience of nursing staff.

Participants tolerate neglectful activities such as omitting showering or social activities, which resonates with research demonstrating that staff “defend” their omissions by downplaying certain care activities to make them less relevant as examples of low care quality. This serves to retain their self-image as caring and compassionate nurses, in line with cognitive dissonance theory [10]. Furthermore, here are practices reflecting ageistic attitudes, which might also confirm this theory in line with prior research finding that negative stereotypes of aging may affect the quality of care accordingly [59]. Intentional or not, this handling of cognitive dissonance and moral distress, depicts neglectful behaviour as less severe, and thus easier for the staff to face in their everyday work. This is also indicated by previous research showing that nursing staff regularly fail to recognise their own practices as neglectful, normalising missed care as a way of legitimising neglect [45]. Tolerating neglect may be a way of enabling existing and insufficient care practices. Intentional or not, tolerance of neglect will indisputably have a negative influence on the quality of care, as well as the well-being of both staff and residents in nursing homes [22, 58, 60].

### Self-protection through avoiding morally distressing situations

Participants further respond to the moral stress related to a neglectful work culture by disengaging emotionally from the caring process. This may reflect further efforts to manage moral distress related to neglectful care provision [50]. Distancing is a well-known coping strategy, as when nursing staff disengage or become detached from a situation to minimise its significance. Not bothering too much enable some of our participants to continue working. This finding is corroborated by research demonstrating that withdrawing emotionally, distancing, and numbing of the conscience are approaches that helps staff to continue working in healthcare [28, 57]. Nevertheless, this avoidance behaviour which initial is a way to mitigate their moral distress also becomes a source of guilt and despair, bringing the personal and professional long-term effects of this coping mechanism into question [28].

Our participants have experienced colleagues who regularly disengage emotionally and physically from their care duties. We cannot know whether this observed behaviour is intentional or not. Distancing from direct patient care may, however, be an intentional way of avoiding morally distressing situations [28]. It has also been shown that a lack of awareness of moral or ethical dilemmas may be a way of handling moral distress, as when staff do not recognise a moral event. This is confirmed in our study by examples of staff refusing to reflect or discuss their own care practices. This may be a way of protecting themselves from moral distress through distancing and/or hardening their emotions [10]. Other research confirms that avoidance of discussion about situations causing moral distress can influence quality of care negatively [25].

Heavy workloads and time pressure have been demonstrated to create emotional and physical stress among nursing staff [16, 26, 50]. Distress, exhaustion, and avoidance (of care) have also been associated with absence from work [57] and intention to quit [16]. This raises concern at a time when there is an increased need for residential care for an increasingly aging population, and difficulties in recruiting skilled nursing staff in Norwegian nursing homes [22]. It has previously been concluded that unfavourable working conditions are the strongest predictors of Norwegian nurses wishing to leave elderly care [26]. Other researchers have found that work overload may not be directly linked to staff turnover and intention to quit, but to role-conflict and ambiguity leading to moral distress [16]. However, this is compounded by research confirming that full withdrawal is a response to moral distress [28]. Our participants verify both these outcomes when they have chosen to retreat from care as a way of protecting both themselves and residents from the burden of neglectful care. For some, this is directly related to the excessive workload, making working in accordance with their own values impossible.

A worrisome finding in our study is participants describing the quality of care in nursing homes as being locked in a “downward spiral” and their concern for the future of care provision. Other researchers have found that nursing staff are leaving their jobs to escape the increasing stress related to losing confidence in their ability to promote sufficient resident safety and quality of care [61]. This may be intended as a constructive approach, to protect both themselves and the residents from neglectful care. On the other hand, the staff who stay, despite their dissatisfaction, may be confined to a role where they are unable to influence the neglectful work culture in a positive way [62].

### Strengths and limitations

The Covid-19 lockdown affected the recruitment process in this study, as nursing home staff were less available and gathering for focus group discussions was no longer an option. This reduced our ability to work towards a true theoretical sampling, to be able to saturate our categories and to provide a grounded theory. However, we managed to reach participants for member-checking, thus strengthening our results. We also managed to recruit a diverse sample of participants from a variety of nursing homes, and we were able to reach former staff who had quit working in nursing homes. Our research team consists of researchers from different disciplines, providing a broader perspective on the theme and possibilities for diverse interpretations of our results.

## Conclusion

Despite that much of the care provision in nursing home is of good quality and resident-centred, a growing body of evidence shows that many nursing home residents’ basic care needs are neglected, and residents do not receive qualitatively good care. This challenges nursing staff´s professional and personal ideals and standards for care and may contribute to moral distress. Our study brings new knowledge on how nursing home staff`s attempt to mitigate their moral distress related to neglectful care practices. We interpret our results as representing a shift from a resident-centred to a staff-centred work culture, where nursing home staff feel compelled to facilitate self-protecting care strategies to make their workday bearable and liveable. This strongly indicates a compromise in the quality of care that enables the continuation of neglectful care practices in Norwegian nursing homes. Finding ways of breaking a downward spiralling quality of care are thus a major concern following our findings. This should be of great interest for managers and policy makers giving the structural and organizational premises for care provision, but also for those being most affected by neglectful care provision such as nursing home resident and their caregivers. Facilitating better working conditions and work culture for caregivers in nursing homes may alleviate the sources creating moral distress.

### Electronic supplementary material

Below is the link to the electronic supplementary material.

Supplementary Material 1

Supplementary Material 2

## Data Availability

The datasets used and/or analysed during the current study are available from the corresponding author on reasonable request.

## Declarations

### Authers’ information

SBL (She/her/hers), Research Fellow, RN. LM (She/her/hers), Professor, MD. WKM (She/her/hers), Professor, PhD, RN. JAS (He/him/his), Professor, PhD, Licensed Psychologist. SBL is doing this research as part of her dissertation research. JAS have contributed with his experience in qualitative research. WKM and LM have long experience in the research field of elder abuse and neglect.

## Abbreviations

A: Assistant
CGT: Constructivist grounded theory
FG: Focus group
II: Individual interview
LPN: Licensed practical nurse
NSD: The Norwegian Centre for Research Data
RCC: Resident-centred care
RN: Registered nurse
SE: Social educator
SW: Social worker
