# Supplementary material for: “Just pee in the diaper” - a constructivist grounded theory study of moral distress enabling neglect in nursing homes
Source: BMC Geriatr. 2024 Apr 24;24:366. doi: 10.1186/s12877-024-04920-7 (PMC11040955; doi:10.1186/s12877-024-04920-7)
Supplement: Supplementary file 1 — Supplementary Material 1 [file 12877_2024_4920_MOESM1_ESM.docx]

# Interview-guide

## Main question

- What regulate nursing staff’ handling of neglect, and how are deviations from this sanctioned?

Introduce case studies or questions from survey-instrument if delayed response or difficulties to understand the topic.

## Follow-up questions

- How do you believe/think neglect should be handled at your workplace?
- What is the culture regarding neglect of residents' needs?
- Which norms (unwritten guidelines) for neglect apply at your workplace?
- What happens if you do not follow these norms at your workplace?
- What is the most challenging about following the norms/not following the norms at your workplace?

Revised interview guide

- Who decides what is good care in your workplace?
- How do you relate to this?
